# A novel pseudoderivative-based mutation operator for real-coded adaptive genetic algorithms

**DOI:** 10.12688/f1000research.2-139.v2

**Published:** 2013-11-19

**Authors:** Maxinder S Kanwal, Avinash S Ramesh, Lauren A Huang

**Affiliations:** 1 Thomas Jefferson High School for Science and Technology, Alexandria, VA 22312, USA

**Keywords:** genetic algorithms, mutation rate, artificial intelligence, bioinformatics, genomics

## Abstract

Recent development of large databases, especially those in genetics and proteomics, is pushing the development of novel computational algorithms that implement rapid and accurate search strategies. One successful approach has been to use artificial intelligence and methods, including pattern recognition (e.g. neural networks) and optimization techniques (e.g. genetic algorithms). The focus of this paper is on optimizing the design of genetic algorithms by using an adaptive mutation rate that is derived from comparing the fitness values of successive generations. We propose a novel pseudoderivative-based mutation rate operator designed to allow a genetic algorithm to escape local optima and successfully continue to the global optimum. Once proven successful, this algorithm can be implemented to solve real problems in neurology and bioinformatics. As a first step towards this goal, we tested our algorithm on two 3-dimensional surfaces with multiple local optima, but only one global optimum, as well as on the N-queens problem, an applied problem in which the function that maps the curve is implicit. For all tests, the adaptive mutation rate allowed the genetic algorithm to find the global optimal solution, performing significantly better than other search methods, including genetic algorithms that implement fixed mutation rates.

## Introduction

The last few years have seen an exponential increase in the size of databases, especially those in genetics, which catalog the basis of various diseases. Computing power has not kept pace with this rapid increase in bioinformatics. Therefore, it has become critical to develop clever algorithms to reduce the time needed to search such databases and arrive at solutions to the treatment of genetically determined diseases ^1^. Genetic algorithms provide new hope to overcome this dilemma. Genetic algorithms attempt to copy the principle of “survival of the fittest”, using Darwin’s theory of evolution to find a satisfactory solution. In essence, a group of random solutions is created and ranked, after which the best solutions are allowed to “interbreed”. During interbreeding, small mutations are allowed to add an element of randomness, and in this way aid the genetic algorithm in finding the optimal solution ^2^.

The applications of genetic algorithms (GAs) are well known and far ranging ^3^. Because of their versatility, GAs have been widely and successfully used to optimize practical problems such as determining a long sequence of base pairs in a genetic database, scheduling drilling operations in a circuit-board factory, and data mining, among others ^4^. However, one major problem faced by GAs is premature convergence, in which the algorithm becomes trapped in a local optimum and is unable to find the global optimum ^5^.

This study focuses on the use of mutations in GAs. Normally, the mutations occur at a constant rate, known as the mutation rate. However, use of a fixed mutation rate can produce sub-optimal solutions ^5^. We propose a new, variable mutation rate that uses a pseudoderivative to take into account the time that a GA can be stuck at a certain point. The longer the algorithm has been stuck at a local optimum, the more likely it is that a mutation will occur. This addition of a greater element of randomness to the algorithm allows it to move from the local optimum and look for better solutions. In this paper, we show that the proposed variable mutation rate outperforms fixed mutation rates and other common search algorithms in the effectiveness of their solutions.

## Background

### Rationale

Many real-life problems can be modeled as continuous, nonlinear optimization problems. Within a given search space *S* on the optimization function *f*, a global (absolute) optimum is sought. This may take the form of a global maximum or minimum, depending on the original problem. A typical 3-dimensional global optimization problem follows the form:

maximize/minimizef(x,y),where(x,y) ∈S    (1)

The challenge in solving a global optimization problem is in seeking the global optimum rather than becoming trapped in a local optimum, an issue that will be addressed in more detail later ^6^. These optimization problems can be approached with a variety of techniques. One popular technique is the use of GAs, the focus of this study ^7^.

### Genetic algorithms (GAs)

GAs are population-based optimization techniques favored for their properties of self-learning, self-organization, self-adaptation, and implicit parallelism ^8^. Based on the principles of Charles Darwin’s natural selection and meiotic cell division, GAs involve several components: a population, a measure of fitness, and a method of breeding ^2^. The population forms the basis for the GA and is made up of many individuals, often called chromosomes. Over time, “chromosomes” breed with other “chromosomes” to form “children” that make up a new generation in the GA ^9^.

Chromosomes can be made up of binary strings or of real values. Binary-coded GAs (BCGAs) have chromosomes of 0s and 1s. While BCGAs are the more traditional method and are adequate for small- to moderate-size optimization problems, they fail for high-dimensional problems because they require more computational time and memory. Real-coded GAs (RCGAs) utilize real values that allow for both improved computational time and memory as compared to BCGAs, making the optimization of multi-dimensional and high-precision problems more feasible ^5^. Each chromosome (binary-coded or real-coded) has a certain fitness value or *f* value derived from its binary string or its real values, where the *f* value of a chromosome refers to its value when inputted into the function described in (1). The calculations for the *f* value of a chromosome vary by problem. In this respect, each chromosome represents a single solution to the optimization problem ^10^. Following the principles of natural selection, chromosomes with higher fitness values give rise to children with high fitness values, so the GA “selects” for fitter chromosomes by giving them a higher probability of breeding and passing on their genes ^11^.

**Figure 1.**
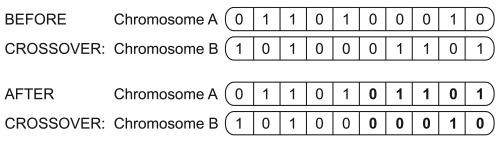
Example of crossover between two binary-coded chromosomes. An example of crossover between a set of two binary-coded chromosomes where half the length of chromosome **A** is exchanged for half the length of chromosome **B**, resulting in a hybrid set of chromosomes.

The breeding stage involves two processes: crossover and mutation. In true meiotic cell division (the process by which a sperm or an egg cell is created), a crossover occurs when two chromosomes pair with each other and exchange portions of their length, forming two hybrid chromosomes. Similarly, chromosomes in a GA exchange values ^12^. A simple example of crossover is illustrated in Figure 1.

The second operator of the breeding stage is mutation. Mutation is a naturally-occurring phenomenon that may come into play during any replication of chromosomes. Incorporation of mutation into a GA may be considered an optional step, but has generally been found to increase the diversity of the population ^13^. This allows GAs to explore promising areas of the search space ^8^. The major advantage of implementing mutations in a GA is in avoiding premature convergence, in which the GA becomes trapped in a local optimum ^14^. However, the mutation operator has trade-offs in slowing down the learning process ^5^. Mutations have in the past taken the form of point, frame-shift, and translocation mutations, all of which involve swapping, switching, or shifting binary bits, in the case of binary-coded chromosomes ^15^. Random mutation has also been proposed, in which a gene is replaced with a random value within certain lower and upper bounds ^16^, and may provide interesting insight into the mechanisms of mutation, since a small mutation in nature may cause a gene to take on a vastly different role ^17^. Finally, the rate of mutation, *r _m_*, can play a key role in the effectiveness of a given GA ^7^.

A variety of modifications have been made to GAs in the breeding stage. A large amount of attention has been devoted to developing improvements in crossover operators, but there have been fewer studies in improving mutation operators, which make up a very promising although largely unexplored branch ^5, 15^. Several studies have implemented adaptive mutation, or a changing mutation rate ^18, 19^. Adaptive mutation methods have ranged from individual gene-based mutations to Gaussian mutation operators based on the mean and the standard deviation of the Gaussian distribution ^20^. This study proposes a novel adaptive mutation approach and applies it to two tests: the well-known N-queens problem and the maximization of a 3-dimensional function. This study also compares a GA implementing an adaptive mutation operator with other well-known search algorithms, such as the Nelder-Mead method, hill climbing technique, and random search.

### Other search algorithms

Numerous other search algorithms besides GAs exist for solving similar optimization problems ^21^. A basic method is the random search method whereby solutions are randomly chosen and evaluated for a certain amount of time, and the best solution found during the time span is returned ^22^. A more advanced method implements the hill climbing technique. This method begins at a random point and uses a greedy algorithm to move to the best immediate neighboring solution until no neighboring solution is better than the current solution ^23^. The final, and most robust, non-GA search algorithm being examined in this study is the Nelder-Mead algorithm. The Nelder-Mead method is a heuristic search that relies on approximating a local optimum for a problem with N variables by using a simplex (a polytope consisting of N + 1 vertices) ^24^.

### The N-queens problem

The N-queens problem is an interesting application for testing the accuracy of genetic algorithms due to the inherent difficulty of optimizing the problem under efficient time and memory constraints ^25^. Given an N × N board, find a set of N-queens such that zero pairs of queens are in the same row, column, or diagonal (none of the queens are attacking each other). Although there are several approaches (brute force/recursion, simulated annealing, etc.) to solving this optimal placement problem ^26^, the N-queens problem was chosen in this study as an early practical test of the proposed new mutation rate operator’s accuracy.

### Optimizing a 3-dimensional function

Finding the maximum or minimum value of a 3-dimensional surface is a more abstract but more visual problem. A surface with multiple local maxima and minima can simultaneously test a GA’s ability to avoid premature convergence and its ability to handle multidimensional optimization problems. Because GAs solve problems with implicit functions of N-dimensions, it is important that the newly proposed mutation operator can deliver reasonable speed and accuracy in the most primal form of the N-dimensional problem, optimizing a space function. While the N-queens problem is adequate for testing the accuracy of a GA, it is not adequate for testing its speed of convergence because there are multiple solutions. Therefore, maximization of a 3-dimensional surface with multiple local maxima but only one absolute maximum was chosen as a practical test of the new mutation operator’s speed of convergence, or number of generations needed to converge to the optimal solution. Furthermore, minimization of the Rastrigin function was chosen as a simple method for comparing accuracies of various search algorithms. The Rastrigin function is an optimal function to choose for such a comparison as the surface holds a myriad of local minima, but only one global minimum at *f* (0,0) = 0. The Rastrigin function models a difficult problem to optimize, where a non-robust search algorithm may easily become caught in a local optimum ^27^.

## Methods

### Experimental design

The research question this study attempted to answer was: what are the effects of an adaptive mutation rate, based on the derivative of the fitness function with respect to generations, on the efficiency and accuracy of a GA? We performed three experiments to answer this question. The first experiment in this study tested the null hypothesis that given a problem, which is not always solvable (to the global optimum) by a fixed-rate GA, a GA with an adaptive mutation rate (based on the derivative of the fitness function with respect to generations) would find an optimal solution significantly more often than one with a constant mutation rate. To reject the null hypothesis, we will need to show that an adaptive mutation rate can find an optimal solution significantly more often than one with a fixed mutation rate. The second experiment tested the null hypothesis that given a problem, which is always solvable (to the global optimum) by a constant mutation rate GA, there would be no significant difference in the efficiency between the constant mutation rate GA and a GA with an adaptive mutation rate, based on the derivative of the fitness function with respect to generation. The third experiment tested the null hypothesis that a GA with an adaptive mutation rate is not significantly more accurate in converging to a global optimum of a 3-dimensional function as compared to other search algorithms (i.e. Nelder-Mead, hill climbing, random).

The mutation rate function *r _m_* was the independent variable in the first study. Accuracy (how often a GA finds the optimum solution) measured in percent (%) with an error value of ±0.001, and efficiency (how long it takes to converge to the solution), measured in generations, were the dependent variables. The experiment was set up with three levels: a constant *r _m_* of 0% (no mutation, control), a constant *r _m_* of 20% (control), and an adaptive *r _m_* derived from a pseudoderivative and a sigmoid function. The independent variable of the second study was the specific search algorithm being run. The accuracy, measured in percent (%) with an error value of ±0.001, was the dependent variable. The experiment was set up with four levels (search algorithms): a random search, a search implementing the hill climbing technique, a search implementing the Nelder-Mead method, and a search implementing the proposed adaptive GA.

The adaptive mutation rate operator was tested on three problems: the N-queens problem, the maximization of a 3-dimensional surface, and the minimization of a different 3-dimensional surface (the Rastrigin function). The N-queens problem was used specifically to test the accuracy of the new adaptive mutation rate since a GA implementing a constant mutation rate does not have 100% accuracy in the N-queens problem. The maximization of a 3-dimensional surface problem was used to test the efficiency of the new adaptive mutation rate since a GA implementing the 20% constant mutation rate does have 100% accuracy in the 3-dimensional maximization problem. The minimization of the Rastrigin function was used to determine the robustness of the proposed adaptive GA compared to other well-known search algorithms. The Rastrigin function was selected due to a most diverse topography, including a myriad of local optima by which weak search algorithms may be forced to prematurely converge. In the first study, accuracy was defined to be the rate of successful optimization within 100,000 generations to the nearest ±0.001, in order to save computing power. Python 2.5 was used to create and run GAs for the N-queens and 3-dimensional maximization problems. Python 3.0 was used to create and run an adaptive GA, Nelder-Mead, hill climbing, and random search algorithms for the Rastrigin function minimization problem. A vector class was created to aid in simplifying the code for the latter experiment. Both GAs reported mutation rate, convergence, and best *f* values for later analysis. For both problems, the mutation rate operator function was a sigmoid function fit to the domain *x* in [0, ∞) and range *y* in [0, 1]. The sigmoid function is defined as:

$$sigmoid\;(x)=\frac{1}{1+e^{-x}}\;(2)$$

with domain *x* in (-∞, ∞), range *y* in [0, 1], and *sigmoid*(0) = 0.5. The function used was thus a fit of the monotonic sigmoid function to the desired domain and range, resulting in

$$r_{m}=2*\left(sigmoid\left(x\right)–0.\text{5}\right)\;(3)$$

where *x* is given by:

$$x=g_{c}–g_{o}\;(4)$$

with *g _c_* representing the current generation and *g _o_* representing the oldest generation of the same best *f* value. *x* thus is inversely related to the derivative of the fitness function with respect to generation. This causes an increase in mutation rate when the fitness between generations is stagnant. A theoretical basis and further details of the adaptive mutation rate operator is included in the Theory section under the Discussion.

For the N-queens problem, the specific case of N = 8 was chosen. The fitness function was chosen for this problem to be the number of pairs of queens violating the problem specification. A program was written to run 200 trials of GAs maxed at 100,000 generations or until a solution was found ( *f* = 0). The genetic code for each solution in the solution set was a sequence of eight integers, with each index representing a column and each value representing a row. Crossover locations were determined using random integers. All variables were held constant apart from mutation rates, which varied between constant mutation at 0% (no mutation), constant mutation rate of 20%, and the sigmoidally-determined adaptive mutation. For the full code, see Script 1 in the Supplementary materials.

**Figure 2.**
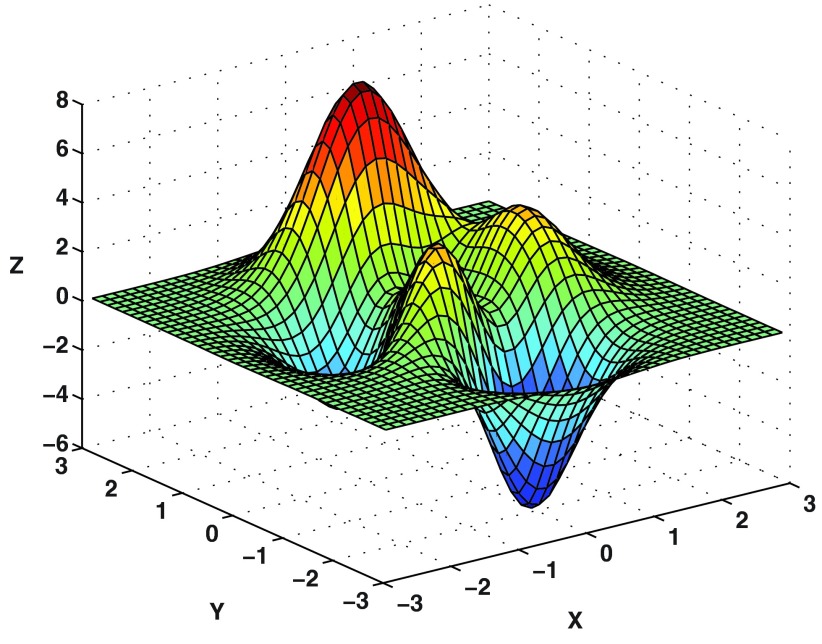
Graphical representation of the surface used for the 3D maximization problem. Visualized in MATLAB 6.5 Using the Command peaks(40). The three maximums of the surface were located at *f* = 8.1165 (global maximum), 3.5507, and 3.4652.

Regarding the maximization of a 3-dimensional function, the proposed sigmoidal mutation operator was again compared to controls of a constant mutation rate of 20% and of no mutation (0%). The function chosen for maximization was

$$z=3*{\left(1-x\right)}^{2}*\;e^{-x^{2}-{\left(y+1\right)}^{2}}\;-10*\left(\frac{x}{5}-x^{3}-y^{5}\right)*e^{-x^{2}-y^{2}}\;-\frac{1}{3}*e^{-{\left(x+1\right)}^{2}-y^{2}}\;(5)$$

which represents a function with several local maxima and minima, which can be solved by the 20% mutation rate GA but not the 0% mutation rate GA. This allowed for a test of efficiency of the new sigmoidally-determined adaptation mutation rate GA against the 20% fixed mutation rate GA and a test of accuracy for the sigmoidally-determined adaptation mutation rate GA against the 0% fixed mutation rate (no mutation) GA. A graphical representation of the function is shown as Figure 2. The genetic code for each solution in the solution set for this problem was a sequence of two integers, the x and y coordinates. The chromosomes were implemented as real-coded chromosomes containing the actual coordinates rather than binary strings. For crossover, the x coordinate of one solution was chosen and the y coordinate of another solution was chosen. For mutation, a coordinate was replaced by a random number within the domain of the problem. Once again, all variables, except for mutation rates, were held constant throughout each experiment.

**Figure 3.**
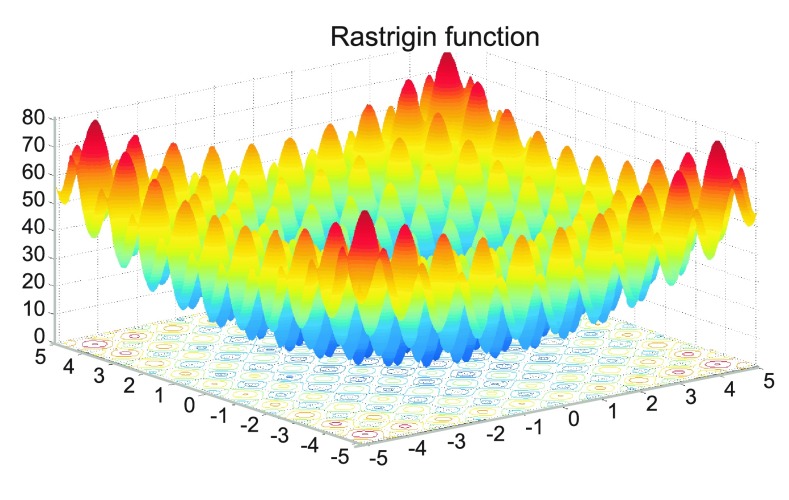
Graphical representation of the Rastrigin function used in the comparison of the Nelder-Mead, Hill Climbing, and Random Search algorithms with the adaptive genetic algorithm. Visualized in MATLAB 6.5: f = @(x,y) 10*2 + x.^2 + y.^2 – 10*cos(2*pi*x) – 10*cos(2*pi*y). The global minimum of the Rastrigin function is *f*(0,0) = 0.

**Figure 4.**
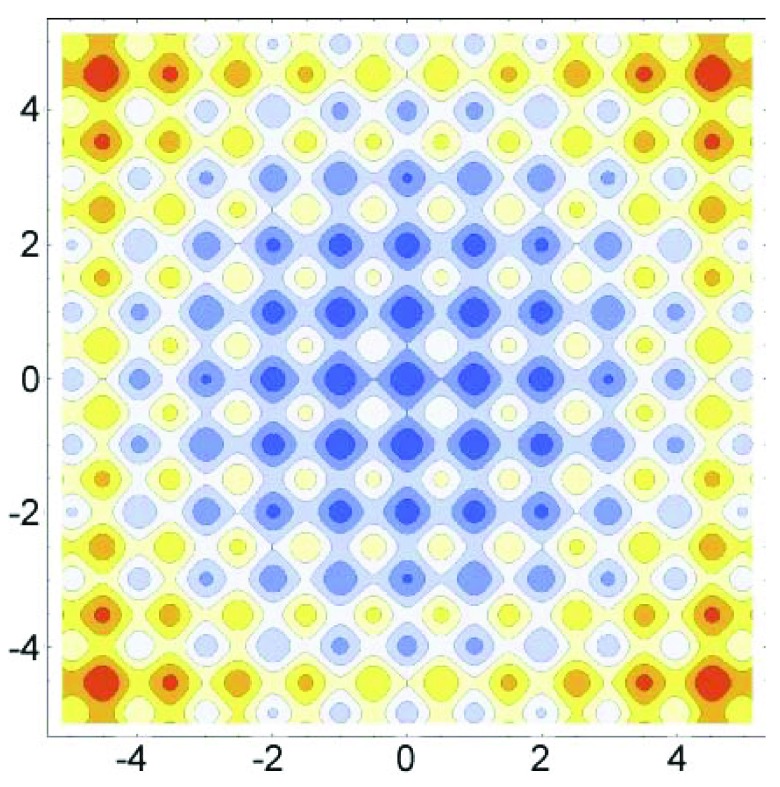
Contour plot of the Rastrigin function. Visualized in Mathematica 8: ContourPlot[10*2 + x^2 + y^2 – 10 Cos[2 Pi x] – 10 Cos[2 Pi y], {x, -5.12, 5.12}, {y, -5.12, 5.12}, ImageSize -> 1000, Axes -> False, ColorFunction -> ColorData["TemperatureMap"]].

The minimization of the Rastrigin function, given by:

$$z=20+x^{2}-10\cos\left(2\pi x\right)+y^{2}-10\cos\left(2\pi y\right)\;(6)$$

was the final test run, with a goal to learn how the proposed adaptive GA compares to non-GA search algorithms. All of the search algorithms were coded to report the run time to converge and the result of the respective convergence. In order for a result to be considered accurate, the returned answer had to be within 0.001 of the true answer. This ensures that the algorithm is converging to the correct minimum and also aids in simulating a difficult optimization problem. Since the random method does not converge, but rather runs for a given time span, the average run time for each GA trial was obtained and used for the run time of the random method search. This allows for direct comparison of the accuracies of the random method and the adaptive GA, as the run time for each method was identical, and thus, held constant. Each algorithm was run for 1,000 trials in order to obtain enough data for a robust analysis. For the full code, see Scripts 2, 3, and 4 in the Supplementary materials. A graphical representation of the Rastrigin function can be found as Figure 3 and Figure 4.

## Results

**Figure 5.**
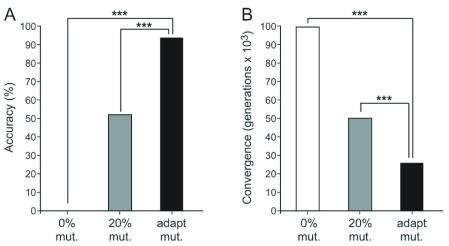
N-queens accuracy and convergence results for constant and adaptive mutation rate genetic algorithms. **A.** Graph of the N-queens problem accuracies among 0% constant mutation, 20% constant mutation, and adaptive mutation. ***p < 0.001. **B.** Graph of the N-queens problem convergences among 0% constant mutation, 20% constant mutation, and adaptive mutation. ***p < 0.001. mutation (mut.), adaptive (adapt.).

The results of experimentation are summarized in Table 1, Table 2, Table 5, and Table 7. In the N-queens problem, the proposed sigmoid mutation operator displayed both the fastest convergence and greatest accuracy with convergence after an average of 25,455.4 generations and an accuracy of 95.5%. In the 3-dimensional surface maximization problem, there was no significant difference between the speeds of the successful GAs. Accuracies were tied between 20% constant and adaptive mutation, as both techniques consistently identified the global maximum within the 100,000-generation limit. In the Rastrigin function minimization problem, the proposed sigmoid mutation operator GA performed significantly better than the three other search algorithms in terms of accurately converging to the global minimum. See Figure 5– Figure 7 for a summary of the results and significant differences.

**Table 1.** Summary of results from solving the N-Queens problem using various mutation rates.

	Constant (0%) mutation	Constant (20%) mutation	Adaptive mutation
**Overall accuracy (%)**	0	53.5	95.5
**Average** **convergence** **(generations)**	100000	50661.49	25455.4

**Table 2.** Summary of results from solving the 3-dimensional maximization problem using various mutation rates.

	Constant (0%) mutation	Constant (20%) mutation	Adaptive mutation
**Overall accuracy (%)**	0	100	100
**Average** **convergence** **(generations)**	100000	67460.99	67779.92

**Figure 6.**
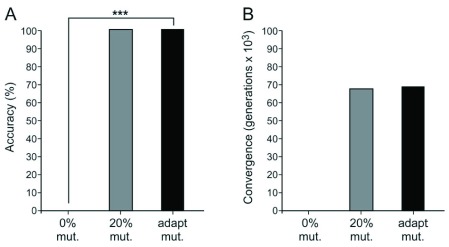
3D maximization accuracy and convergence results for constant and adaptive mutation rate genetic algorithms. **A.** This is a graph of the 3D surface maximization problem accuracies among 0% constant mutation, 20% constant mutation, and adaptive mutation. **B.** This is a graph of the 3D surface maximization problem convergences among 20% constant mutation and adaptive mutation. 0% constant mutation was not statistically analyzed because convergence was not achieved. ***p < 0.001. mutation (mut.), adaptive (adapt.).

**Figure 7.**
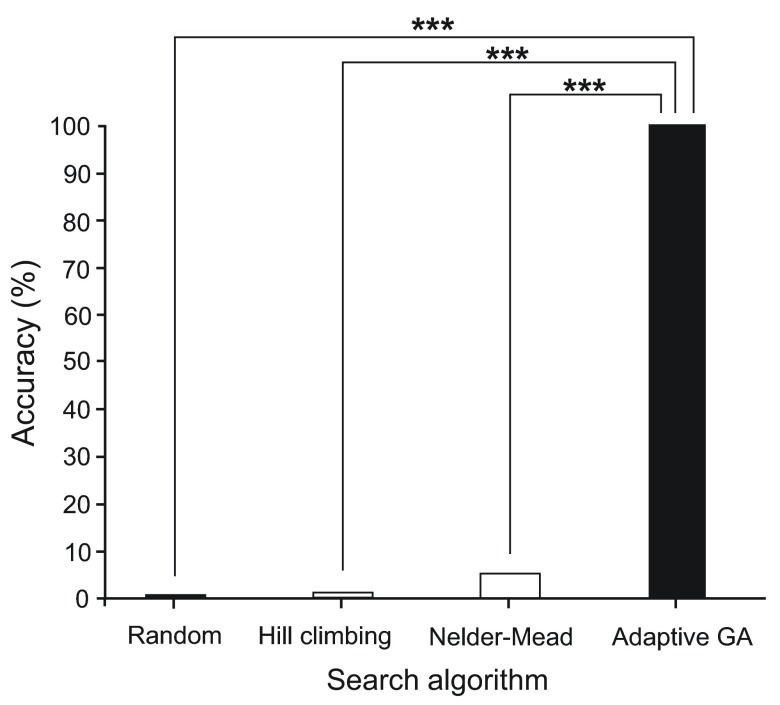
Rastrigin function minimization accuracy results for various search algorithms. Graph of the Rastrigin function minimization problem accuracies among random, Hill climbing, Nelder-Mead, and adaptive GA methods. ***p < 0.001. genetic algorithm (GA).

### N-queens

In the N-queens problem, a series of unpaired, single-tailed Student’s t-tests were used to test the following null hypothesis at the 0.05 level of significance: the accuracy of the adaptive mutation operator will not be significantly higher than that of 20% constant mutation or that of 0% constant mutation. The null hypothesis was rejected for the 20% constant mutation rate (p = 6.108 × 10 ^-25^ < 0.05) and the 0% constant mutation rate (p = 2.572 × 10 ^-214^ < 0.05). See Table 3 for a summary of the N-queens statistics.

**Table 3.** Summary of statistical tests on N-queens results comparing the accuracies and convergence generations associated with adaptive and constant mutation rates.

T-tests	p-value
Constant (0%) mutation vs. adaptive mutation – accuracy	2.572 × 10 ^-214^
Constant (0%) mutation vs. adaptive mutation – convergence	6.2864 × 10 ^-137^
Constant (20%) mutation vs. adaptive mutation – accuracy	6.10806 × 10 ^-25^
Constant (20%) mutation vs. adaptive mutation – convergence	1.86354 × 10 ^-10^

Accuracies, run times, and statistics for genetic algorithms with various mutation rates solving the N-queens problem. In the nqueensnomut, nqueensconstmut, and nqueenssigmoid tabs, the end heuristic (number of queens attacking each other on the board, with 0 m) found by that genetic algorithm is displayed along with the time the algorithm took to converge to that solution (in generations). In the stats tabs, t-tests are run to compare the accuracies and convergence generations among the different mutation rate genetic algorithms. Key: mut rate: mutation rate, converg: convergence (generations), end h (end heuristic) nomut: no mutation, constmut, constant mutation.

### Maximizing a 3-dimensional function

In the surface maximization problem, a series of unpaired, single-tailed Student’s t-tests were used to test the following null hypothesis at the 0.05 level of significance: the efficiency of the 20% constant mutation rate GA will be significantly higher than that of the adaptive mutation operator GA. The null hypothesis was rejected (p = 0.448 > 0.05) for the 20% level. Because the constant 0% mutation rate GA does not find the global maximum, it was compared to the adaptive mutation operator on the basis of null hypothesis 1 at the 0.05 level of significance: the accuracy of the adaptive mutation operator will not be significantly higher than that of 0% constant mutation rate. The null hypothesis was rejected (p = 0 < 0.05). See Table 4 for a summary of the surface maximization statistics.

**Table 4.** Summary of statistical tests on 3-dimensional maximization results comparing the accuracies and convergence generations associated with adaptive and constant mutation rates.

T-tests	p-value
Constant (0%) mutation vs. adaptive mutation – accuracy	0
Constant (0%) mutation vs. adaptive mutation – convergence	1.1566 × 10 ^-56^
Constant (20%) mutation vs. adaptive mutation – accuracy	0.5
Constant (20%) mutation vs. adaptive mutation – convergence	0.44759

Accuracies, run times, and statistics for genetic algorithms with various mutation rates maximizing a 3D function. In the 3dfloatnomut, 3dfloatconstmut, and 3dfloatsigmoid tabs, the maximum value found by that genetic algorithm is displayed along with the time the algorithm took to converge to that maximum (in generations). In the stats tabs, t-tests are run to compare the accuracies and convergence generations among the different mutation rate genetic algorithms.

### Minimizing the Rastrigin function

In the Rastrigin function minimization problem, a series of unpaired, single-tailed Student’s t-tests were used to test the following null hypothesis at the 0.05 level of significance: the accuracy of the adaptive mutation operator GA will not be significantly higher than those of the other search methods (Nelder-Mead, hill climbing, random). The null hypothesis was rejected for random method (p = 2.53 × 10 ^-223^ < 0.05), for the hill climbing method (p = 9.61 × 10 ^-268^ < 0.05), and for the Nelder-Mead method (p = 3.04 × 10 ^-152^ < 0.05). Furthermore, a series of unpaired, single-tailed Student’s t-tests were used to test if there were significant differences in the run times of the hill climbing and Nelder-Mead methods when the algorithm accurately converged and when the same algorithm did not accurately converge. Both the hill climbing method (p = 0.140 > 0.05) and the Nelder-Mead method (p = 0.576 > 0.05) showed no significant differences in run time when accurately converging compared to inaccurately converging. These results point to the possibility that these two methods were only accurate when a fortunate random point was selected to start running the search algorithm from. No extra time being needed to converge correctly versus incorrectly, points towards the idea that no extra computation was needed either. See Table 6 and Table 7 for a summary of the Rastrigin function minimization statistics.

**Table 5.** Summary of results for solving the Rastrigin function minimization problem using various search algorithms.

	Random	Hill climbing	Nelder-Mead	Adaptive genetic algorithm
**Accuracy (%)**	0.2	0.8	5.7	100
**Average run time to correctly converge (s)**	0.27405	0.65205	0.01852	0.27405
**Average converged minimum value**	0.49505	16.97789	4.76441	0
**Trials**	1000	1000	1000	1000

**Table 6.** Summary of statistical tests on the Rastrigin function minimization results comparing the accuracies associated with each search algorithm.

T-tests	p-value
Random vs. adaptive genetic algorithm – accuracy	2.53 × 10 ^-223^
Hill climbing vs. adaptive genetic algorithm – accuracy	9.61 × 10 ^-268^
Nelder-Mead vs. adaptive genetic algorithm – accuracy	3.04 × 10 ^-152^

**Table 7.** Summary of results and statistics comparing run times for accurate and inaccurate convergences using the Hill climbing and Nelder-Mead methods.

	Convergence	Hill climbing	Nelder-Mead
**Run time (s)**	Accurate	0.65205	0.01852
	Inaccurate	0.59429	0.01830
**Trials**	Accurate	35	57
	Inaccurate	4965	943
**p-value**		0.13960	0.57576

Accuracies, run times, and statistics for search algorithms minimizing the Rastrigin function. In the Nelder-Mead Results, Hill Climber Results, Random Search, and Genetic Algorithm tabs, the minimum value found by that method is displayed along with the time the algorithm took to arrive at that minimum. In the Statistics and Graph tabs, t-tests are run to compare the accuracies of three search algorithms to the adaptive genetic algorithm.

## Discussion

### Theory

The mutation rate operator presented in this study uses randomness to guide a genetic algorithm (GA) towards the optimal solution. A GA with less randomness leads to faster convergence towards local optima; however, by limiting randomness it limits the search space, which in turns hinders the search for the global optimum. Conversely, a GA with more randomness hinders progress towards local optima, but allows for a wider search space, aiding the search for the global optimum ^8, 28^. Therefore, randomness should be inversely related to the derivative of the fitness function *f*. Because *f* is implicit in most problems, the proposed heuristic attempted to look at the derivative of *f* with respect to generation, creating a pseudo, inversely related function to the derivative that could be used to calculate an adaptive mutation rate. The heuristic subtracted the first generation at which the most optimal fitness value appeared from the current generation to obtain a result inversely related to the derivative, and thus directly related to the randomness or mutation rate. Therefore any monotonically increasing function with domain bounded by *x* in [0, ∞) and *y* in [0, 1] should provide an appropriate amount of randomness to either find a local optimum or increase the search space from the local optima in order to find the global optimum.

### Implications

The implications of a new, pseudoderivative-based adaptive mutation rate are considerable. The implementation of this new technique within a genetic algorithm could provide increased accuracy in optimization of all GA problems. For example, the treatment of complex diseases requires the discovery of new drug combinations that are hard to come by solely on the basis of empirical clinical knowledge. Search algorithms, including GAs, have begun to provide promising results in identifying optimal drug combinations, e.g. for destroying human cancer cells as well as for minimizing the physiological decline associated with aging. Moreover, these approaches required only one-third of the number of tests employed in the classic method for the discovery of optimal drug combinations. This approach has greatly reduced the risk and expense of clinical trials ^29^. The availability of a more sophisticated class of GAs will also allow search algorithms that are based on GAs to be improved. For example, swarm-based optimization algorithms, such as the Bees Algorithm, can improve the efficiency with which optimal and suboptimal solutions can be discovered within a given search space ^30^.

Our results agree with results reported in other articles on adaptive mutation accuracy and efficiency ^5, 31^. The method described in this study also has the added benefit of low computational complexity while still being able to guide the GA out of local optima and towards the global optimum. Studies in adaptive mutation have ranged widely from individual gene-based mutations, to Gaussian operators, to polynomial operators ^20^. However, to our knowledge, past adaptive mutation techniques have never used the derivative of *f* with respect to generation to determine a changing mutation rate. This new method of implementing adaptive mutation may open up entirely new areas for implementing search algorithms based on improved GAs.

## Conclusion

GAs are powerful tools that can optimize overwhelmingly complex real-life problems, including speedy diagnoses of complex diseases. Previous GAs faced a trade-off between speed and accuracy, as more random GAs sacrificed speed in exchange for a better chance of optimization or less random ones gave up accuracy for speed. The solution of an adaptive mutation operator based on the derivative of *f* with respect to generation allows for increased accuracy without the loss of speed. Suggestions for research that would expand upon current findings include determining an optimal monotonically increasing function for the mutation operator, such as a sigmoid, inverse tangent, or scaled linear function, as well as comparing the current GA to other commonly used search methods in current bioinformatics problems.

## Supplementary materials

Genetic algorithm scripts Script 1: genetic algorithm for the N-Queens problem. Script 2: Genetic algorithm for the Rastrigin function optimization problem. Script 3: Nelder-Mead, Hill Climbing, and Random Search algorithms for the Rastrigin function optimization problem. Script 4: The vector class created to simplify the Nelder-Mead, Hill Climbing, and Random Search algorithms for the Rastrigin function optimization problem.
