# Supplementary material for: A novel pseudoderivative-based mutation operator for real-coded adaptive genetic algorithms
Source: F1000Res. 2013 Nov 19;2:139. Originally published 2013 Jun 11. [Version 2] doi: 10.12688/f1000research.2-139.v2 (PMC3924953; doi:10.12688/f1000research.2-139.v2)
Supplement: Genetic algorithm scripts — Script 1: genetic algorithm for the N-Queens problem. Script 2: Genetic algorithm for the Rastrigin function optimization problem. Script 3: Nelder-Mead, Hill Climbing, and Random Search algorithms for the Rastrigin function optimization problem. Script 4: The vector class created to simplify the Nelder-Mead, Hill Climbing, and Random Search algorithms for the Rastrigin function optimization problem. [file f1000research-2-2353-s0003.tgz › Script_1.docx]

from random import *

from math import *

from sys import *

import pdb

# handle command line arguments for board dimensions

if len(argv) > 1:

n = int(argv[1])

else:

n = 8

max = n * (n - 1) / 2

def show(board):

print

for r in range(n):

s = ""

for c in range(n):

if (r + c) % 2 == 1:

s += chr(27) + "[31;47"

else:

s += chr(27) + "[31;45"

s += "m"

if board[c] == r:

s += chr(27) + "[1m" + "X"

else:

s += " "

print chr(27) + "[0m" + s + chr(27) + "[0m"

print chr(27) + "[0m"

def calc_h(board): #fitness function

h = 0

for x in range(1,len(board)): #loop through columns

for y in range(x+1,len(board)): #loop through columns > x

if board[x] == board[y] or abs(board[x] - board[y]) == abs(x - y): #if queens are attacking add 1 to h

h+=1

return h

def main():

for i in range(200): #200 trials

best_h = -100 #best h value, h <=0

bg = 0 #first generation of the best h value

board = [] #population of solutions

for x in range(2*n): #2n boards

board.append([]) #represents a board

for k in range(n):

board[-1].append(randint(0, n-1)) #random initialization

board[x].insert(0, calc_h(board[x])*-1) #h-value for random initialization

generations = 0

while generations<100000: #generations capped at 100,000

board.sort() #sort boards by fitness function

if board[-1][0] > best_h: #take board with best (greatest) h-value and compare it to best h-value to date

bg = generations #if greater reset best h_value to date and the generation where it occurs

best_h = board[-1][0]

if board[-1][0] == 0:

show(board[-1][1:])

print board[-1]

print generations+1

print 'Success!'

break

mutation = 2*(1/(1+exp(-(generations-bg))) - 0.5) #sigmoidal adaptive mutation

#mutation = 0.2 #used for constant mutation of 20% level

#mutation = 0 #used for 0 mutation

generations+=1

board = board[len(board)/3:len(board)] #gets rid of the worst third

while len(board) < 2*n: #crossover and mutation

#CROSSOVER

temp1 = board[randint(0, n-1)]

temp2 = board[randint(0, n-1)]

crosspoint = randint(0, n-1)

newboard = temp1[:crosspoint] + temp2[crosspoint:] #child = different parts of parents

#MUTATION

for i in range(n-1): #check for adding mutations

if(random() < mutation):

newboard[i] = randint(0,n-1)

newboard[0] = calc_h(newboard)*-1 #calc_h for child

board.append(newboard)

print i,board[-1][0], bg,mutation

if __name__ == "__main__": main()
