# Supplementary material for: A novel pseudoderivative-based mutation operator for real-coded adaptive genetic algorithms
Source: F1000Res. 2013 Nov 19;2:139. Originally published 2013 Jun 11. [Version 2] doi: 10.12688/f1000research.2-139.v2 (PMC3924953; doi:10.12688/f1000research.2-139.v2)
Supplement: Genetic algorithm scripts — Script 1: genetic algorithm for the N-Queens problem. Script 2: Genetic algorithm for the Rastrigin function optimization problem. Script 3: Nelder-Mead, Hill Climbing, and Random Search algorithms for the Rastrigin function optimization problem. Script 4: The vector class created to simplify the Nelder-Mead, Hill Climbing, and Random Search algorithms for the Rastrigin function optimization problem. [file f1000research-2-2353-s0003.tgz › Script_2.docx]

from random import *

from math import *

from sys import *

from time import clock

import pdb

def calc_h(board):

x = board[1]

y = board[2]

return -(20 + x**2 - 10*cos(2*pi*x) + y**2 - 10*cos(2*pi*y))

def main():

output = open('output_GA.txt', 'w') # output data to txt file

sum = 0

for i in range(1000):

start = clock()

best_h = -10000

bg = 0

n = 20

board = []

for x in range(n):#2n boards

board.append([])#represents a board

board[x].append(0)

board[x].append(random()*10.24-5.12)#random initialization

board[x].append(random()*10.24-5.12)

board[x][0] = calc_h(board[x])#h-value for random initialization

#print board

generations = 0

while True:#generations < 100000: #generations

#print generations,board[0][0],board[-1][0]

board.sort()

if board[-1][0] > best_h:

bg = generations

best_h = board[-1][0]

best_x = board[-1][1]

best_y = board[-1][2]

mutation = (1/(1+exp(-(generations-bg))) - 0.5)

generations+=1

#print generations, board[0][0], board[-1][0], mutation, best_x, best_y

board = board[len(board)/3:len(board)] #gets rid of the worst third

while len(board) < n:#crossover and mutation

#CROSSOVER

temp1 = board[randint(0,len(board)-1)]

temp2 = board[randint(0,len(board)-1)]

newboard = []

newboard.append(1)

newboard.append(temp1[1])

newboard.append(temp2[2])

#newboard[0]=calc_h(newboard)*-1

#MUTATION

if(random() < mutation):

newboard[1] = random()*10.24-5.12

if(random() < mutation):

newboard[2] = random()*10.24-5.12

newboard[0] = calc_h(newboard)

board.append(newboard)

board.sort()

if abs(board[-1][0]) <= 0.001:

end = clock()-start

output.write('1 ' + str(-1*board[-1][0]) + ' ' + str(end) + '\n')

print (-1*board[-1][0]), end

sum += end

break

#mutation,best_x,best_y,board[-1][0], bg

print 'Total Run Time:', sum

output.close()

if __name__ == "__main__": main()
