# Supplementary material for: A novel pseudoderivative-based mutation operator for real-coded adaptive genetic algorithms
Source: F1000Res. 2013 Nov 19;2:139. Originally published 2013 Jun 11. [Version 2] doi: 10.12688/f1000research.2-139.v2 (PMC3924953; doi:10.12688/f1000research.2-139.v2)
Supplement: Genetic algorithm scripts — Script 1: genetic algorithm for the N-Queens problem. Script 2: Genetic algorithm for the Rastrigin function optimization problem. Script 3: Nelder-Mead, Hill Climbing, and Random Search algorithms for the Rastrigin function optimization problem. Script 4: The vector class created to simplify the Nelder-Mead, Hill Climbing, and Random Search algorithms for the Rastrigin function optimization problem. [file f1000research-2-2353-s0003.tgz › Script_3.docx]

from VectorClass import *

def nextTriangle(A,B,C): #The Nelder-Mead algorithm used here to obtain a single new point.

#Case 0 Sort the Vectors

[B,C,A] = Vector.sort([A,B,C]) #B.cost() <= C.cost() <= A.cost()

#Case 1: vertex A moves to D or E.

D = Vector(B + C - A)

E = Vector(3*(B+C)/2 - 2*A)

F = Vector((3*(B+C)-2*A)/4)

G = Vector((2*A + B + C)/4)

H = Vector((A+B)/2)

I = Vector((B+C)/2)

if D.cost() < A.cost() and E.cost() < A.cost():

A.equals(E)

return B,C,A

elif D.cost() < A.cost():

A.equals(D)

return B,C,A

#Case 2: vertex A moves to F or G.

if G.cost() < F.cost():

X = Vector(G)

else: X = Vector(F)

if X.cost() < A.cost():

A.equals(X)

return B,C,A

#Case 3: vertex A moves to H and vertex C moves to I.

else:

A.equals(H)

C.equals(I)

return B,C,A

def triangleHasNotConverged(A, B, C): # Boolean result

if (A-B).mag() < SMALLEST_TRIANGLE_SIZE:

return False

if (A-C).mag() < SMALLEST_TRIANGLE_SIZE:

return False

if (B-C).mag() < SMALLEST_TRIANGLE_SIZE:

return False

return True

def threeRandomVectors(): #The domain is restricted to this problem.

from random import random

A = Vector((random()*2-1)*DOMAIN_LIMIT, (random()*2-1)*DOMAIN_LIMIT)

B = Vector((random()*2-1)*DOMAIN_LIMIT, (random()*2-1)*DOMAIN_LIMIT)

C = Vector((random()*2-1)*DOMAIN_LIMIT, (random()*2-1)*DOMAIN_LIMIT)

return [A,B,C]

def NelderMeadSearch():

from time import clock

START = clock()

A, B, C = threeRandomVectors()

while (triangleHasNotConverged(A, B, C)):

B,C,A = nextTriangle(A,B,C)

[B,C,A] = Vector.sort([A,B,C])

global COUNT

if B.cost() <= (GLOBAL_MIN + ERROR): COUNT+=1

return B.cost()

def RandomSearchBestResult():

from random import random

from time import clock

START = clock()

minCost = float('inf')

while(clock() - START <= GA_RUN_TIME):

A = Vector((random()*2-1)*DOMAIN_LIMIT, (random()*2-1)*DOMAIN_LIMIT)

if A.cost() < minCost:

minCost = A.cost()

global COUNT

if minCost <= (GLOBAL_MIN + ERROR): COUNT+=1

return minCost

def HillClimberHasNotConverged(best, lastStep):

if (best.cost() - lastStep.cost()) == 0:

return False

return True

def HillClimberSearch():

from time import clock

from random import random

from math import sin

from math import cos

from math import pi

A = Vector((random()*2-1)*DOMAIN_LIMIT, (random()*2-1)*DOMAIN_LIMIT)

startTime = clock()

lastStep = 100000*A # Initially set to arbitrarily high vector

while (HillClimberHasNotConverged(A, lastStep)):

best = A

lastStep = best

for rad in range (1,33): # Find best movement by sampling 32 equally spaced points on a circle (r = 0.001) around A

B = A + Vector(.001*cos(rad*2*pi/32), .001*sin(rad*2*pi/32))

if B.cost() < best.cost():

best = B # Store best movement

A = best # Make A the best step

global COUNT

if best.cost() <= (GLOBAL_MIN + ERROR): COUNT+=1

return best.cost()

def CorrectConvergence(result): # Boolean (0 or 1) returning if algorithm converged correctly

if result <= (GLOBAL_MIN + ERROR): return 1

return 0

#GLOBAL CONSTANTS

SMALLEST_TRIANGLE_SIZE = 0.001 # minimum length of any side

DOMAIN_LIMIT = 5.12 # -DL <= x <= DL and -DL <= y <= DL, DL = DOMAIN_LIMIT

GLOBAL_MIN = 0.000 # True Global Minimum

ERROR = 0.001

COUNT = 0 # Number of accurate convergences (error = +/- .01)

TRIALS = 1000 # Number of trials run for each search algorithm

GA_RUN_TIME = 0.274047756097561 # Average run time per Correct GA Convergence

def main():

from time import clock

output = open('output_NelderMead.txt', 'w') # output data into a txt file

for i in range(TRIALS): # Number of Trials

START = clock()

result = NelderMeadSearch()

runTime = clock() - START

correct = CorrectConvergence(result)

output.write(str(correct) + ' ' + str(result) + ' ' + str(runTime) + '\n')

output.close()

output = open('output_HillClimber.txt', 'w') # output data into a txt file

for i in range(TRIALS): # Number of Trials

START = clock()

result = HillClimberSearch()

runTime = clock() - START

correct = CorrectConvergence(result)

output.write(str(correct) + ' ' + str(result) + ' ' + str(runTime) + '\n')

output.close()

output = open('output_Random.txt', 'w') # output data into a txt file

for i in range(TRIALS): # Number of Trials

START = clock()

result = RandomSearchBestResult()

runTime = clock() - START

correct = CorrectConvergence(result)

output.write(str(correct) + ' ' + str(result) + ' ' + str(runTime) + '\n')

output.close()

global COUNT

COUNT = 0

for i in range(TRIALS):

print('SEARCH RESULST:')

print('1. Nelder-Mead\t\tcost=', NelderMeadSearch())

print('2. Random Search\tcost=', RandomSearchBestResult())

print('3. Hill climber\t\tcost=', HillClimberSearch())

print('\nNumber of Accurate Convergences: ', COUNT, '\n')

if __name__== '__main__': main()
