# Supplementary material for: A novel pseudoderivative-based mutation operator for real-coded adaptive genetic algorithms
Source: F1000Res. 2013 Nov 19;2:139. Originally published 2013 Jun 11. [Version 2] doi: 10.12688/f1000research.2-139.v2 (PMC3924953; doi:10.12688/f1000research.2-139.v2)
Supplement: Genetic algorithm scripts — Script 1: genetic algorithm for the N-Queens problem. Script 2: Genetic algorithm for the Rastrigin function optimization problem. Script 3: Nelder-Mead, Hill Climbing, and Random Search algorithms for the Rastrigin function optimization problem. Script 4: The vector class created to simplify the Nelder-Mead, Hill Climbing, and Random Search algorithms for the Rastrigin function optimization problem. [file f1000research-2-2353-s0003.tgz › Script_4.docx]

*Script 4: The Vector Class created to simplify the Nelder-Mead, Hill Climbing, and Random Search Algorithms for the Rastrigin Function Optimization Problem.*

class Vector(object):

def __init__(self, *data):

if type(data[0])==Vector:

self.data = list(data[0])

else:

self.data = list(data)

def __repr__(self):

return repr(tuple(self.data))

def print(self, n=0):

print("Vector(", end ="")

for j in range(len(self.data)-1):

print (round(self.data[j],n), end = ", ")

print (round(self.data[j+1],n), end = "")

print(") ")

return self

#--FUNCTIONS--------------------------------------------------------

def length(self):

return (len(self.data))

def scalars(self):

print('scalars =', self.data[:])

return self.data[:]

def vectorList(self):

return self.data

def equals(self, other):

self.data=other.vectorList()

return tuple(self.data)

def vecCost(x,y):

from math import cos

from math import pi

if (-5.12<=x<=5.12) and (-5.12<=y<=5.12):

return (20+ x**2 - 10*cos(2*pi*x) + y**2 - 10*cos(2*pi*y))

return float('inf')

def cost(self):

from math import cos

from math import pi

if (-5.12 <= self.data[0] <= 5.12) and (-5.12 <= self.data[1] <= 5.12):

x = self.data[0]

y = self.data[1]

return (20+ x**2 - 10*cos(2*pi*x) + y**2 - 10*cos(2*pi*y))

return float('inf')

def dist(self, other):

return (self-other).mag()

def dotProd(self, other):

return sum([self.data[j]*other.data[j] for j in range (len(self.data))])

def crossProd(X,Y):

return Vector (X.data[1]*Y.data[2] - X.data[2]*Y.data[1], \

X.data[2]*Y.data[0] - X.data[0]*Y.data[2], \

X.data[0]*Y.data[1] - X.data[1]*Y.data[0])

def mag(self):

from math import sqrt

return sqrt(sum([j*j for j in self.data]))

def normalize(self):

m=self.mag()

self.data= (self/m).data

return self

def swap (A,B):

T = Vectors(0)

T.equals(A)

A.equals(B)

B.equals(T)

def sort(vectorList):

#if type(vectorList) != list or len(vectorList) != 3 or type(vectorList[0]) != Vector or type(vectorList[1] != Vector or type(vectorList[2]) != Vector:

#exit('Error: The sort function limited to a list of three Vector elements.')

vectorList.sort(key = Vector.cost)

return vectorList

#--OPERATORS--------------------------------------------------------

def __add__(self, other):

return Vector(*[self.data[j]+other.data[j] for j in range(len(self.data))])

def __sub__(self, other):

return -other + self

def __mul__(self, entity):

if isinstance(entity, (Vector)):

return self.crossProd(entity)

if isinstance(entity, (int, float)):

return Vector(*[ j*entity for j in (self.data) ] )

def __rmul__(self, num):

return self*num

def __truediv__(self, num):

if num==0: return NotImplemented

return self *(1.0/num)

def __eq__(self, other):

return (self.data == other.data)

def __ne__(self, other):

return not(self.data == other.data)

def __neg__(self):

return Vector(*[-j for j in self.data])

def __getitem__(self, index):

return self.data[index]

def __setitem__(self, index, num):

self.data[index]=num

return self
