# Supplementary material for: A novel pseudoderivative-based mutation operator for real-coded adaptive genetic algorithms
Source: F1000Res. 2013 Nov 19;2:139. Originally published 2013 Jun 11. [Version 2] doi: 10.12688/f1000research.2-139.v2 (PMC3924953; doi:10.12688/f1000research.2-139.v2)
Supplement: Peer review history

# Peer review: Referee response for version 2

**DOI:** 10.5256/f1000research.2353.r2563

**Published:** 2014-03-12

**Authors:** Christos Ouzounis

**Affiliations:** 1 Computational Genomics Unit and Institute of Agrobiotechnology, National Center for Research & Technology, Thessalonica, Greece

The authors have addressed many of the concerns raised.

I have read this submission. I believe that I have an appropriate level of expertise to confirm that it is of an acceptable scientific standard.

# Peer review: Referee response for version 2

**DOI:** 10.5256/f1000research.2353.r2562

**Published:** 2014-02-10

**Authors:** Panayiotis (Takis) Benos

**Affiliations:** 1 Department of Computational Biology, University of Pittsburgh, Pittsburgh, PA, USA

I have read this submission. I believe that I have an appropriate level of expertise to confirm that it is of an acceptable scientific standard.

# Peer review: Referee response for version 2

**DOI:** 10.5256/f1000research.2353.r2561

**Published:** 2013-11-27

**Authors:** Mark Ragan

**Affiliations:** 1 Institute for Molecular Bioscience, University of Queensland, Brisbane, Australia

The authors have addressed my concerns, particularly, in regards to the wording of the title.

I have read this submission. I believe that I have an appropriate level of expertise to confirm that it is of an acceptable scientific standard.

# Peer review: Referee response for version 1

**DOI:** 10.5256/f1000research.1167.r1179

**Published:** 2013-07-22

**Authors:** Panayiotis (Takis) Benos

**Affiliations:** 1 Department of Computational Biology, University of Pittsburgh, Pittsburgh, PA, USA

This article primarily focuses on a very interesting computational problem, namely whether the variable-rate mutation operators in a genetic algorithm offers an advantage over the fixed-rate ones. The main idea is that variable-rate operators will help algorithms escape the local optima and identify the global optimum more efficiently. The authors indeed show that this is the case in at least some of the examples they investigated. I think this is a very promising article that will generate a lot of interest in this area.

However, I am a bit unsure about the method’s relevance to biology. There are some biological examples that I can think of where their methodology can make an impact but I fail to see how it can be used for “diagnosis” and “therapy” as the title states. I wish they had provided some more examples towards that direction.

Finally, I would like to bring up a point that is frequently overseen in computational studies. Although in many non-biological problems finding the optimal solution is the ultimate goal, many biological problems are better served by finding a set of optimal+ sub-optimal solutions. Examples include protein and RNA folding, and microRNA and transcription factor binding sites. Perhaps the authors would consider extending their methodology towards that direction in the future.

I have read this submission. I believe that I have an appropriate level of expertise to confirm that it is of an acceptable scientific standard, however I have significant reservations, as outlined above.

# Response

**Published:** 2013-11-19

**Authors:** Maxinder Kanwal

**Affiliations:** Thomas Jefferson High School for Science and Technology, USA

We agree with the reviewer’s point. Our main objective was to show the advantage variable-rate mutation operators in genetic algorithms had over fixed-rate ones when applied correctly. While some of our immediate visions for the algorithm were for applying it towards solving problems in bioinformatics, we realize that the algorithm is more of a general search method that can be applied to a number of problems not just within biology. In order to more accurately portray the content of this article, the title and abstract have been modified to avoid emphasizing a minor aspect of the article. In addition, the Implications section of the Discussion have been expanded on to more comprehensively explain how this algorithm can be applied in the medical field as an example of one of its many applications.

In regards to your comment, you bring up a very good point. We will certainly look into modifying this algorithm to make it a technique that better serves attempting to solve problems in biology by having it search for a set of optimal and suboptimal solutions.

# Peer review: Referee response for version 1

**DOI:** 10.5256/f1000research.1167.r1062

**Published:** 2013-07-15

**Authors:** Mark Ragan

**Affiliations:** 1 Institute for Molecular Bioscience, University of Queensland, Brisbane, Australia

Genetic algorithms offer a biologically motivated approach to nonlinear optimisation. In the so-called breeding stage of a genetic algorithm, the diversity of candidate solutions is increased by operations analogous to crossover between chromosomes, and point mutations. If parameterised appropriately, the mutation operator helps the genetic algorithm explore the fitness landscape, in particular by making it less likely to become trapped in a local optimum. Mutation operators are typically of fixed rate, although there is some prior art in the use of variable-rate operators. Here Kanwal, Ramesh and Huang, senior students at Thomas Jefferson High School for Science and Technology in Alexandria, Virginia, develop some theory on the form of adaptive-rate mutation operators, and investigate the performance of a new heuristic motivated by this theory. They report that this heuristic delivers much-improved accuracy in finding the global optimum, at no cost in speed, in two reasonably difficult optimisation scenarios, and describe bounds on the mutation function.

The research is clearly of publishable quality. The content explained clearly and in sufficient detail to allow the work to be replicated, and the conclusions are balanced and justified on the basis of the results presented. The writing style is exemplary. However, the manuscript does not deliver against the “diagnosis and therapy” promised in the title and abstract: indeed, the authors rightly allude to much broader contexts. In the absence of a specific case study in the biological application domain, the title might focus solely on the algorithmic contribution. It would be interesting to compare the performance of genetic algorithms implementing their new mutation operator with, for example, simulated annealing or multi-chain MCMC, although this would presumably require a broader comparison framework than was within-scope for the work reported here.

Figures 3 and 4 are very similar to those in Wikipedia (http://en.wikipedia.org/wiki/Rastrigin_function, accessed 13 July 2013); does this arise from use of a generic parameterisation?

I would prefer “optima” rather than “optimums”.  There is a misspelling in the legend of Figure 3.

I have read this submission. I believe that I have an appropriate level of expertise to confirm that it is of an acceptable scientific standard.

# Response

**Published:** 2013-11-19

**Authors:** Maxinder Kanwal

**Affiliations:** Thomas Jefferson High School for Science and Technology, USA

We agree with the reviewer’s point. Our main objective was to show the advantage variable-rate mutation operators in genetic algorithms had over fixed-rate ones when applied correctly.  While some of our immediate visions for the algorithm were for applying it towards solving problems in bioinformatics, we realize that the algorithm is more of a general search method that can be applied to a number of problems not just within biology. In order to more accurately portray the content of this article, the title and abstract have been modified to avoid emphasizing a minor aspect of the article. In addition, the Implications section of the Discussion have been expanded on to more comprehensively explain how this algorithm can be applied in the medical field as an example of one of its many applications.

In regards to Figures 3 and 4 being similar to those on Wikipedia, the reason for this is because the code given below the images was almost the same code used to generate our figures in the paper with only slight modifications to make the figures more readable.  We were aware of the Wikipedia images before generating our own; we simply liked the parameters set in the Wikipedia code, and so we generated our images using that code (along with some additional style changes).

The mistakes pointed out have been fixed as well.

# Peer review: Referee response for version 1

**DOI:** 10.5256/f1000research.1167.r1057

**Published:** 2013-07-12

**Authors:** Christos Ouzounis

**Affiliations:** 1 Computational Genomics Unit and Institute of Agrobiotechnology, National Center for Research & Technology, Thessalonica, Greece

Genetic algorithms (GAs) represent an adaptive computation technique with applications to various optimization problems, borrowing ideas from biological systems – with solution populations and operators that mimic DNA mutation and selection. As the authors of this report mention, GAs can suffer from issues of convergence to local optima, as all optimization algorithms do. It is proposed that a variable mutation rate (as opposed to the more classical approach of a constant mutation rate) can contribute towards better optimization performance, away from local optima: the longer the GA remains at a local optimum, the higher the mutation rate can become. This is the key idea that is addressed in this work.

The study is interesting as it focuses on the mutation operator which has not been researched sufficiently, compared to the crossover operator which has been studied quite extensively. The proposed solution - namely a variable mutation rate as an independent variable, at least in one case - is compared to other optimization techniques, such as random search / hill climbing / Nelder-Mead. Experiments are performed for well-established, highly controlled optimization problems, such as the N-Queens problem, maximization of a 3D surface and the Rastrigin function. Performance in terms of both accuracy of solution and efficiency of resource consumption was monitored for all experiments. Interestingly, while adaptive mutation has a two-fold performance in both terms above for the N-Queens problem, there is no significant difference for the 3D surface problem.

This is interesting work, but relevance to computational biology and real-world solutions is somewhat peripheral. I am sure the authors are investigating applications of their approach to larger problems, as alluded in their title - which unfortunately is somewhat misleading: without the terms ‘diagnosis’ and ‘therapy’, this would have been a fine, technical report on an exciting subject. Hopefully, we will see some further development towards that direction in the near future.

I have read this submission. I believe that I have an appropriate level of expertise to confirm that it is of an acceptable scientific standard, however I have significant reservations, as outlined above.

# Response

**Published:** 2013-11-19

**Authors:** Maxinder Kanwal

**Affiliations:** Thomas Jefferson High School for Science and Technology, USA

We agree with the reviewer’s point. Our main objective was to show the advantage variable-rate mutation operators in genetic algorithms had over fixed-rate ones when applied correctly. While some of our immediate visions for the algorithm were for applying it towards solving problems in bioinformatics, we realize that the algorithm is more of a general search method that can be applied to a number of problems not just within biology. In order to more accurately portray the content of this article, the title and abstract have been modified to avoid emphasizing a minor aspect of the article. In addition, the Implications section of the Discussion have been expanded on to more comprehensively explain how this algorithm can be applied in the medical field as an example of one of its many applications.
